# Enhanced FGF21 Delivery via Neutrophil-Membrane-Coated Nanoparticles Improves Therapeutic Efficacy for Myocardial Ischemia–Reperfusion Injury

**DOI:** 10.3390/nano15050346

**Published:** 2025-02-23

**Authors:** Zhiheng Rao, Yuli Tang, Jiamei Zhu, Zhenzhen Lu, Zhichao Chen, Jiaojiao Wang, Yuxuan Bao, Alan Vengai Mukondiwa, Cong Wang, Xiaojie Wang, Yongde Luo, Xiaokun Li

**Affiliations:** 1 School of Pharmaceutical Sciences, Wenzhou Medical University, Wenzhou 325035, China; logoswzmc@gmail.com (Z.R.); yuriitang@163.com (Y.T.); zjm15727965307@163.com (J.Z.); lzzlzz0213@163.com (Z.L.); czc3622@163.com (Z.C.); erjiao626@163.com (J.W.); baoyuxuan233@163.com (Y.B.); alanvengaimk@gmail.com (A.V.M.); wangcong0814@163.com (C.W.); susanwang1214@wmu.edu.cn (X.W.); 2 Oujiang Laboratory, Wenzhou 325000, China; 3 The First Affiliated Hospital of Wenzhou Medical University, Wenzhou 325000, China

**Keywords:** neutrophil-membrane-coated nanoparticle, fibroblast growth factor 21 (FGF21), drug delivery, ischemia and reperfusion injury, myocardial infarction

## Abstract

Acute myocardial infarction, a leading cause of death globally, is often associated with cardiometabolic disorders such as atherosclerosis and metabolic syndrome. Metabolic treatment of these disorders can improve cardiac outcomes, as exemplified by the GLP-1 agonist semaglutide. Fibroblast growth factor 21 (FGF21), a novel metabolic regulator, plays pivotal roles in lipid mobilization and energy conversion, reducing lipotoxicity, inflammation, mitochondrial health, and subsequent tissue damage in organs such as the liver, pancreas, and heart. Here, we test the therapeutic efficacy of FGF21 in mice with ischemia–reperfusion (I/R) injury, a model of acute myocardial infarction. We employed the strategic method of coating the FGF21-encapsulating liposomal nanoparticles with a neutrophil membrane designed to camouflage FGF21 from macrophage-mediated efferocytotic clearance and promote its targeted accumulation at I/R foci due to the inherent neutrophilic attraction to the inflammatory site. Our findings revealed that the coated FGF21 nanoparticles markedly accumulated within the lesions with a prolonged half-life, in additional to the liver, leading to substantial improvements in cardiac performance by enhancing mitochondrial energetic function and reducing oxidative stress, inflammation, and cell death. Therefore, our research highlights a viable strategy for the enhanced delivery of therapeutical FGF21 analogs to lesions beyond the liver following myocardial infarction.

## 1. Introduction

Acute myocardial infarction (AMI) remains a life-threating disease with a high mortality rate [1]. The cause of AMI is multifactorial, primarily due to underlying coronary artery disease, often complicated by cardiometabolic disorders such as atherosclerosis, diabetes, obesity, and hyperlipidemia. Reperfusion therapy is the most effective approach for AMI. However, it frequently results in myocardial ischemia and reperfusion (I/R) injury, which unintentionally leads to extended cardiomyocyte death and worsens AMI and associated heart pathologies [2]. The mechanisms underlying I/R injury may include oxidative stress, inflammation, mitochondrial dysfunction, and cardiomyocyte apoptosis [3,4,5], leading to heart failure and patient death [2,3,6,7,8]. Therefore, novel effective pharmacotherapies are urgently needed [9,10,11,12,13].

Fibroblast growth factor 21 (FGF21) is a stress-inducible polypeptide hormone that plays a crucial role in maintaining metabolic and energy homeostasis. It promotes lipid catabolism, fatty acid oxidation, glucose disposal, and heat dissipation of energy stores in an endocrine manner [14,15,16,17,18]. This helps counteract stress-induced metabolic derangements and associated tissue inflammatory and oxidative damage. In the cardiovascular system, administered FGF21 ameliorates cardiomyopathy, hypertrophy, and ischemic damage by inhibiting lipid accumulation, oxidative stress, inflammation, endothelial dysfunction, and cardiomyocyte apoptosis [19]. Therefore, FGF21 holds potential as a therapeutic strategy for metabolic diseases and associated tissue dysfunction, including heart diseases. Long-acting FGF21 analogs have entered phase 3 clinical trials for patients with MASH and fibrosis. However, due to the poor pharmacodynamic and pharmacokinetic properties, diffusive systemic effect, and short serum half-life of FGF21 and its analogs, developing an effective targeted delivery strategy for FGF21-based long-acting drugs will significantly enhance therapeutic potential [20].

Local inflammation and immune cell infiltration are common early stages of tissue damage, with neutrophils being the predominant immune cells to arrive at the site. This phenomenon can be leveraged for enhanced drug delivery. In this study, we developed an approach for the targeted delivery of pharmacological FGF21 to the I/R site by embedding it in neutrophil-membrane-coated nanoparticles [21]. Our results demonstrate that this approach effectively enriches FGF21 at the I/R foci beyond the liver, which is a common post-injection drug metabolizing site, leading to enhanced anti-inflammatory and antiapoptotic effects, as well as improved cardiac function.

## 2. Materials and Methods

### 2.1. Reagents and Antibodies

All reagents used in this study were obtained from commercial sources. RPMI-1640 medium and FBS were purchased from Invitrogen (Carlsbad, CA, USA). The purified recombinant endotoxin-free human FGF21 (rhFGF21) was obtained from an internal source (Wenzhou Medical University, Wenzhou, China) [22]. Anti-Bcl-2, anti-p65, and anti-phospho-p65 antibodies were obtained from Zhenbio (Chengdu, China). Anti-IKK, anti-phospho-IKK, anti-IKB, anti-phospho-IKB, and anti-HO-1 antibodies were obtained from Cell Signaling Technology (Danvers, MA, USA). Anti-BAX, anti-ERK, and anti-phospho-ERK antibodies were obtained from Proteintech (Wuhan, China). Texas red and ICG (Indocyanine green) were obtained from Thermo Fisher Scientific (Waltham, MA, USA). Alexa Fluor 488 and 594 were obtained from Yeasen (Shanghai, China). Cell Counting Kit-8 was from Beyotime (Nantong, China).

### 2.2. Animal Study

The animal experimental protocol was approved by the Wenzhou Medical University Animal Care and Use Committee and was conducted in accordance with the National Institutes of Health Guide for the Care and Use of Laboratory Animals. The healthy wildtype male C57BL/6J mice (6–8 weeks old; 20–25 g) used in this study were purchased from Slac Laboratory Animal Corporation (Shanghai, China) and housed under controlled humidity (50 ± 10%) and temperature (25 °C ± 2 °C) conditions with a 12/12 h light/dark cycle, with ad libitum access to a standard chow diet and water.

### 2.3. Myocardial Ischemia–Reperfusion Injury Model

The I/R mouse model was established in C57BL/6J mice (20–25 g, 6–8 weeks old). The mice were anesthetized with isoflurane (0.33 mL/min at 4 L/min fresh gas flow) via orotracheal intubation connected to a ventilator. The heart was exposed after left thoracotomy between the fourth and fifth intercostal spaces. The left anterior descending artery was ligated with a 7-0 silk suture, and ischemia was allowed for 30 min. The slipknot was then released, and the myocardium was perfused for 72 h. The mice were randomly grouped to receive the following treatments intraperitoneally: PBS (300 μL); NP_rhFGF21_ (300 μL, 0.35 mg); NM-NP_rhFGF21_ (300 μL, 0.35 mg). The animals were then sacrificed, and tissues were collected on day 3. As the sham group, another 6 mice received thoracotomy but without left coronary artery ligation.

### 2.4. Preparation of NP_rhFGF21_

The NP_rhFGF21_ was prepared using the liposomes with the extrusion method through vacuum rotary distillation. Briefly, 1.1 mg cholesterol, 10.2 mg DPPC, 2.2 mg DSPC, and 6.5 mg DOPC were added to a certain amount of chloroform in proportion, and the mixture was then evaporated in a water bath at 65 °C for 30 min. Then, 1 mL rhFGF21 at 2 mg/mL in phosphate-buffered saline (PBS) was added to the dry lipid film. The solution was vortexed for 15 s at maximum speed three times to resuspend the lipid materials in the solution and then sonicated in a water bath for 5 min to allow rhFGF21-containing liposome nanoparticles to form.

Two polycarbonate membranes with a pore size of 400 nm were used to extrude the liposome preparation. The liposome suspension was passed through the polycarbonate membranes 20 times on ice until the sample appeared to be semi-translucent. This step was repeated sequentially on 200 nm and 100 nm polycarbonate membranes to obtain small unilamellar liposomes. The formed liposomes were transferred to a storage vial filled with N_2_ or argon gas to reduce lipid oxidation. Excess lipid materials were removed via overnight dialysis against a 8 kDa cutoff membrane in 4 °C. Texas Red and Indocyanine Green (ICG, Thermo Fisher Scientific, USA) were loaded into the NPs at a weight ratio of 0.5% and 2%, respectively, for in vitro and in vivo experiments. The encapsulation efficiency percentage (EE%) of the drug was calculated by the equation (EE%) = [(W_initial rhFGF21_ − W_free rhFGF21_)/W_initial rhFGF21_] × 100%. The loading efficiency of the drug was calculated by the equation (LE%) = [(W_initial rhFGF21_ − W_free rhFGF21_)/W_total NM-NPrhFGF21_] × 100%.

### 2.5. Preparation of Neutrophil Membrane

The neutrophil membrane was prepared using the discontinuous density gradient centrifugation method in the presence of Percoll. Briefly, the tibia and fibula were separated from the C57BL/6J mice (20–25 g; 6–8 weeks old). Both ends of the tibia and fibula were cut off, and the bone cavity was flushed with RPMI-1640 medium until the bone appeared to be pale. Then, 2 mL of cell suspension was slowly added to 55% and 65% Percoll double-layer suspension. After centrifugation at 2500 rpm for 30 min, the neutrophil fraction was collected from the interface between the 55% and 65% Percoll layers. The collected cells were resuspended in TM buffer (50 mM Tris HCl, 10 mM Magnesium Sulfate at pH 7.5) and extruded by passing the suspension through the support membranes 20 times. The cell suspension was added with sucrose to form a 0.25 M solution, which was then centrifuged at 2000 rpm at 4 °C for 10 min. The resulting supernatant was centrifuged at 15,000 rpm for 30 min. The precipitate (cell membrane) was collected.

### 2.6. Preparation and Characterization of NM-NP_rhFGF21_

For constructing the NM-NP_rhFGF21_, the above neutrophil membrane was mixed with nanoparticles in a 300:1 (*w*/*w*) ratio. The mixture was extruded by passing the suspension through 200 nm and 100 nm polycarbonate membranes 20 times, respectively, on ice. The excess cell membrane was removed via dialysis with a 8 kDa cutoff membrane for 2 h.

The size and zeta potential of the nanoparticles were measured using a Zetasizer Nano ZS (Malvern Panalytical, Malvern, UK). The morphology was examined using JEM 1200EX transmission electron microscopy (TEM, JEOL, Tokyo, Japan). For the in vitro release assay, NP_rhFGF21_ and NM-NP_rhFGF21_ were added to 10% FBS medium and incubated for 1, 3, 6, 12, 24, and 72 h at 37 °C. The NPs were then removed using centrifugation. The concentrations of rhFGF21 in solution were determined using an enzyme-linked immunosorbent assay (ELISA, Jialai, Beijing, China) as directed by the manufacturer protocol.

### 2.7. Identification of Membrane-Associated Proteins

The neutrophil lysate, neutrophil membrane, and NM-NPs were lysed on ice for 30 min in NP-40 lysis buffer. The lysates were centrifuged at 12,000× *g* for 30 min. The supernatants were mixed with loading buffer and boiled for 10 min. All samples were loaded onto SDS-PAGE at 30 μg of protein. The gel was stained using a Fast Silver Stain kit (Beyotime, China). For Western blot, the proteins in the gel were transferred to PVDF membranes, which were then blocked with 5% nonfat milk for 1 h at room temperature. Primer antibodies against CD81 (1:1000), Na^+^-K^+^-ATPase (1:1000), IL-1R2 (1:1000), CXCR2 (0.5 μg/mL), and β-actin (1:5000) were incubated with the membranes for 3 h at room temperature before being incubated with secondary antibodies. The protein membranes were processed for visualization in an Amersham Imager 680 following standard protocol (GE, Healthcare, Chicago, IL, USA).

### 2.8. Cellular Uptake of Texas-Red-Labeled NM-NP_rhFGF21_

The mouse macrophage cell line Raw 264.7 was sourced from ATCC and cultured in DMEM containing 10% fetal bovine serum (FBS, Thermo Fisher Scientific, USA) and 1% penicillin/streptomycin in a humid incubator with 5% CO_2_ at 37 °C. Raw 264.7 cells were inoculated in confocal dishes. After 12 h, the cells were washed with PBS and added with fresh serum-free medium containing NP-Texas Red and NM-NP_rhFGF21_-Texas Red (20 μg/mL for Texas Red). After being cultured for 2 h, the cells were fixed and stained with DAPI. Intracellular drug uptake was observed using a Leica-TCS SP8 confocal laser scanning microscope (CLSM, Leica, Wetzlar, Germany).

### 2.9. In Vivo Biodistribution Study

For the in vivo biodistribution analysis, I/R mice were administered with equal volumes of NM-NP_rhFGF21_, ICG-labeled NP_rhFGF21_, and ICG-labeled NM-NP_rhFGF21_ intravenously (*i.v.*). The in vivo images were captured and assessed with the IVIS Spectrum imaging system (Caliper, Princeton, NJ, USA) at 8 h post injection. Excitation and emission were set at 745 nm and 840 nm, respectively. To further investigate the organ distribution of NPs ex vivo, the heart and other major organs (liver, lung, kidney, and spleen) were collected under euthanasia conditions. Each organ’s fluorescence signal was quantitatively assessed by the IVIS Spectrum imaging system.

### 2.10. In Vitro Evaluation of Bioactivity of NM-NP_rhFGF21_

Human embryonic kidney 293T cells (sourced from ATCC) transfected with betaKlotho (KLB)-expressing plasmid were cultured in DMEM containing 10% fetal bovine serum (FBS) and 1% penicillin/streptomycin in a humid incubator with 5% CO_2_ at 37 °C. 293T-KLB cells in 6-well plates with subconfluency (~80%) were switched to FBS-free DMEM for 12 h. After washing with PBS, the cells were stimulated with 100 ng/mL rhFGF21 and NM-NP_rhFGF21_ in fresh serum-free medium for 5 min and then collected for Western blotting. The transferred PVDF membranes were incubated with primer antibodies against ERK1/2 (1:1000) or phospho-ERK1/2 (1:1000) for 3 h at room temperature before being incubated with secondary antibodies. The resulting membranes were then processed for visualization in an Amersham Imager 680 following the standard protocol (GE, Healthcare, USA).

### 2.11. Cellular Toxicity Assessment

The rat cardiomyocyte cell line H9C2 was sourced from ATCC and cultured in DMEM containing 10% fetal bovine serum (FBS, Thermo Fisher Scientific, USA) and 1% penicillin/streptomycin in a humid incubator with 5% CO_2_ at 37 °C. After induction with all-trans retinoic acid, the differentiated H9C2 cells were cultured in 96-well plates. After 12 h, the cells were washed with PBS and incubated with fresh serum-free medium containing NM-NP_rhFGF21_ at 0, 5, and 30 μg/mL. After 6 h incubation, the cells were washed with PBS and added with fresh serum-free medium containing 10% CCK8 (Cell Counting Kit-8, Beyotime, China). After 1 h incubation, cell viability was assessed in a SpectraMax 190 Microplate Reader (Molecular Devices, San Jose, CA, USA) according to the Cell Counting Kit-8 instruction.

### 2.12. Assessment of Cardiac Function

All mice were anesthetized using isoflurane for echocardiography. Echocardiographic parameters were obtained in a Vevo 3100 system with 30 MHz probe (Fujifilm VS, Toronto, ON, Canada). Left ventricular (LV) end-systolic and -diastolic diameters were calculated in 2D M-mode, and the cardiac parameters were analyzed under a double-blind condition. LV fraction shortening (LVFS%) and LV ejection fraction (LVEF%) were then calculated according to LV end-systolic diameter (LVESD) and LV end-diastolic dimeter (LVEDD).

### 2.13. Evaluation of Infarct Size via Pathological Staining

The hearts were collected immediately after echocardiographic measurements for evaluating the injury in the acute phase with 2,3,5-triphenyl tetrazolium chloride (TTC, Solarbio, Beijing, China) staining. The hearts were harvested, frozen at −80 °C for 1 h, and cut into 5 slices below the ligature. The slices were incubated with TTC solution at 37 °C for 30 min without light and fixed in 4% paraformaldehyde for 4 h at room temperature. The images were captured using a digital camera, and the infarct size was analyzed in a double-blind manner with IPP 6.0 software. Stained areas were padded with red and transformed for OD calibration.

### 2.14. Quantitative Real-Time PCR

The total RNA was isolated from mouse tissues using the RNAiso Plus (TaKaRa, Osaka, Japan) kit including DNase treatment. The total RNA at 1 μg was retrotranscribed with a HiScript III 1st Strand cDNA Synthesis kit (Vazyme, Nanjing, China) in the presence of random primers following the manufacturer’s instructions. Two microliters of a 1/10 dilution of the first-strand cDNA mixture were used in each PCR reaction, with a total reaction volume of 10 μL, using the ChamQ Universal SYBR qPCR Master Mix (Vazyme, China). The qPCR was performed in a CFX96 Touch™ Real-Time PCR Detection System Brochure, with the respective gene primers listed in Table 1. After confirming the specificity of the PCR products according to the melting curve, the comparative cycle threshold method was used for calculating the expression levels of genes of interest, using hypoxanthine phosphoribosyltransferase 1 (*Hprt1*) as an internal standard.

### 2.15. ROS Assay

The frozen tissue was cut into 6–8 μm slices. A DCFH-DA lipid peroxidation probe was used to analyze myocardial ROS in accordance with the manufacturer’s instructions. The DCFH-DA solution at 10 mM was added to the tissues and incubated at 37 °C for 30 min. The ROS-positive region was visualized as red fluorescence under an OLYMPUS BX53 fluorescence microscope.

### 2.16. Evaluation of Apoptosis via TUNEL Staining

The frozen tissue was cut into 6–8 μm slices and a terminal deoxynucleotidyl transferase dUTP nick-end labeling (TUNEL) assay was performed using a commercial kit (Roche, Mannheim, Germany). The TUNEL-positive cells and nuclei were visualized as blue and red fluorescence under microscopy, respectively. The apoptotic index was calculated as the ratio of TUNEL-positive cardiomyocytes to total nuclei.

### 2.17. Evaluation of Inflammation by Immunofluorescence

Frozen tissues were cut into slices at 6–8 μm and incubated for 2 h with primary anti-F4/80 antibody at 10 μg/mL. An Alexa Fluor 488-conjugated secondary antibody (1:500) was used for visualization, with green fluorescence representing the macrophages and blue fluorescence representing the nuclei. Images were obtained using an OLYMPUS BX53 fluorescence microscope (Olympus Corporation, Tokyo, Japan).

### 2.18. Western Blotting Analysis

The heart tissues were homogenized and lysed in RIPA buffer (150 mM NaCl, 25 mM Tris–HCl, 1% sodium deoxycholate, 0.1% sodium dodecyl sulfate, and 1% Nonidet P-40) supplemented with phosphatase and protease inhibitors at 4 °C for 30 min. The complexes were then centrifuged at 12,000 rpm at 4 °C for 30 min. The protein concentration in the supernatant was quantified using a BCA kit (Thermo Fisher Scientific, Rockford, IL, USA). The supernatants with 40 ug total protein were mixed with the loading buffer, boiled for 10 min, and then subjected to SDS-PAGE. After transfer, the PVDF membranes were blocked with 5% nonfat milk in TBST for 1 h and then incubated with the following primary antibodies for 12 h at 4 °C: anti-BAX (1:1000), Anti-BCL-2 (1:1000), anti-p65 (1:1000), anti-phospho-p65 (1:1000), anti-IKK (1:1000), anti-phospho-IKK (1:1000), anti-IKB (1:1000), anti-phospho-IKB (1:1000), anti-HO-1 (1:1000), and anti-β-actin (1:5000). The membranes were washed with TBST three times and incubated with the secondary antibody (1:10,000) for 2 h. Signals were visualized using Amersham Imager 680 (GE, Healthcare, USA). The intensity of immunoreactivity was quantified using NIH Image J software (1.54f). All experiments were repeated at least three times.

### 2.19. Statistical Analysis

All data are expressed as means ± standard error of the mean of at least three independent experiments. The two-tailed unpaired Student’s *t*-test was used for a single comparison. One-way analysis of variance was used for multiple group comparisons. In all analyses, *p* < 0.05 was taken to indicate statistical significance.

## 3. Results

### 3.1. Neutrophil Membrane Coating of FGF21 Nanoparticles Is Feasible with Enhanced Slow Release and Half-Life

**Figure 1:**
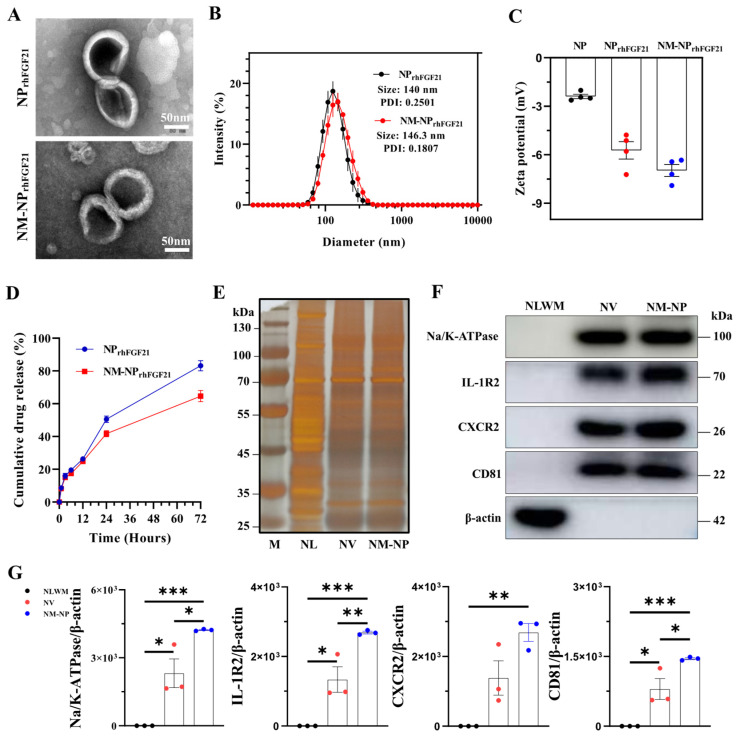
Characterization of NM-NP_rhFGF21_. (**A**) Transmission electron microscope image of NP_rhFGF21_ (rhFGF21 nanoparticles) and NM-NP_rhFGF21_ (neutrophil-membrane-coated rhFGF21 nanoparticles). Scale bar = 50 nm. (**B**) Diameters of NP_rhFGF21_ and NM-NP_rhFGF21_ particles measured by DLS. n = 6. PDI: polydispersity Index. (**C**) Zeta potential of NP, NP_rhFGF21_, and NM-NP_rhFGF21_. n = 4. (**D**) Stability of NM-NP_rhFGF21_ in PBS without 10% FBS over time. n = 3. (**E**) Total protein content visualization of neutrophil lysates (NLs), neutrophil-membrane-derived vesicles (NVs), and neutrophil-membrane-coated NPs (NM-NPs) stained with silver solution. (**F**,**G**) Western blot identification and quantification of neutrophil membrane markers CD81, Na/K-ATPase, IL-1R, and CXCR2 in neutrophil lysates without membrane fraction (NLWM), NV, and NM-NP. n = 3. Each bar represents the mean ± standard error of the mean (SEM), * *p* < 0.05, ** *p* < 0.01, *** *p* < 0.001, between two groups.

Neutrophil-membrane-enveloped nanoparticles (NM-NPs) have been tested as stable and efficient carriers for targeted macromolecular drug delivery [23]. We used the vacuum rotary distillation and extrusion method to first envelop drug-grade recombinant human FGF21 (rhFGF21) into liposome NPs, forming the NP_rhFGF21_ particles. The rhFGF21-carrying liposomes were then coated with purified cell membrane fractions from activated mouse bone marrow neutrophils (Figure S1). Transmission electron microscopy (TEM) images showed a sphere-like unilamellar membrane structure for both NM-NP_rhFGF21_ and NP_rhFGF21_ particles after phosphotungstic acid staining (Figure 1A). Dynamic light scattering (DLS) measurements revealed that the hydrodynamic diameter of NM-NP_rhFGF21_ increased by only ~6.3 nm with a lower PDI value compared to uncoated NP_rhFGF21_, indicating excellent uniformity in particle size and distribution (Figure 1B). The sizes of NM-NP _rhFGF21_ were relatively well-maintained over a period of 3 days (Figure S2), indicating excellent stability for the formed rhFGF21 nanoparticles. The negative surface zeta potential was reduced in NM-NP_rhFGF21_ (Figure 1C). NM-NP_rhFGF21_ in medium containing fetal bovine serum (FBS) that mimics the circulation condition showed a tendency of slower rhFGF21 release (Figure 1D). The encapsulation efficacy was calculated to be 70.2% and 67.8% for NP_rhFGF21_ and NM-NP_rhFGF21_, with a loading capacity of 6.38% and 6.16%, respectively.

Next, we validated the authenticity and transfer efficiency of the neutrophil membrane coating on NM-NP_rhFGF21_. Silver staining demonstrated that both the NM-NP coating and neutrophil membrane vesicle (NV) shared a similar total proteome profile, which was not observed in the neutrophilic lysate (Figure 1E). Immunoblot analysis revealed the presence and enrichment of key neutrophilic surface antigens [24], including CD81, sodium/potassium-transporting ATPase (Na/K-ATPase), IL-1 receptor 2 (IL-1R2), and CXC motif chemokine receptor 2 (CXCR2) on NM-NP compared to that of NV, but not the membrane-depleted fraction (Figure 1F,G). These results indicate a successful coating of NP_rhFGF21_ particles by the plasma membrane isolated from activated neutrophils.

**Figure 2:**
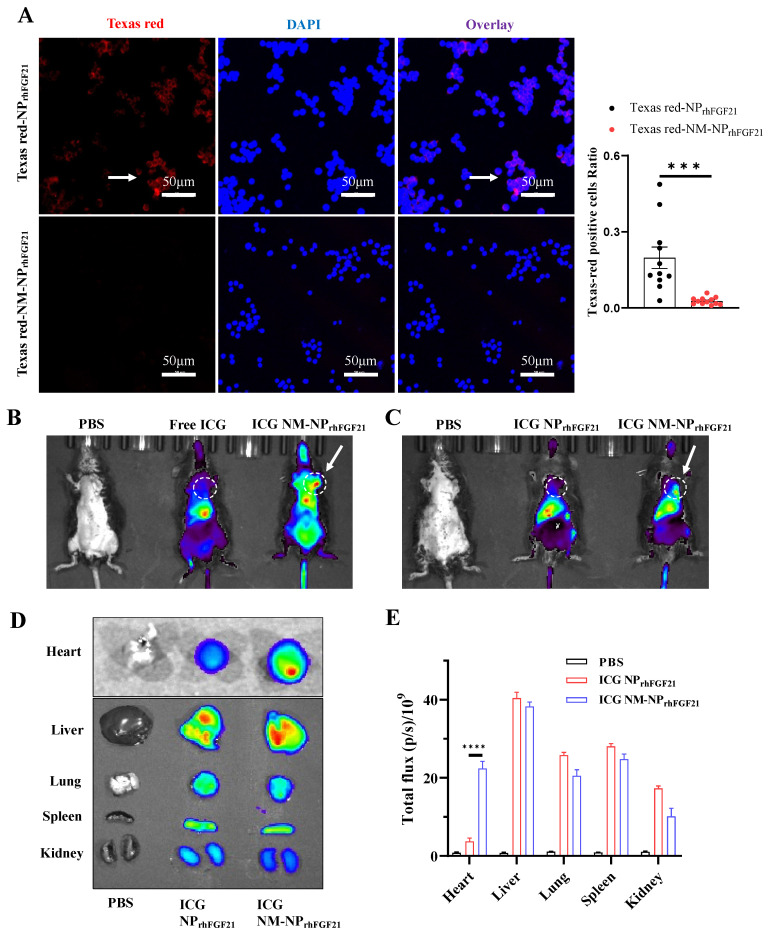
Reduced macrophage-mediated clearance and enhanced heart targeting capacity of NM-NP_rhFGF21_. (**A**) Representative fluorescence images and statistical analysis of RAW 264.7 cells incubated with Texas Red-NP_rhFGF21_ or Texas Red-NM-NP_rhFGF21_ for 6 h. Red represents nanoparticles and blue represents nuclei. Scale bar: 50 μm. n = 11–12. (**B**,**C**) In vivo fluorescence images of I/R injured mice at 6 h post administration of ICG-loaded NM-NP_rhFGF21_ compared to ICG-loaded NP_rhFGF21_ and free ICG. Ex vivo fluorescence images (**D**) and mean radiant efficiencies (**E**) of major organs isolated from the above mice as indicated. Each bar represents the mean ± standard error of the mean (SEM), *** *p* < 0.001, and **** *p* < 0.0001 between two groups.

The natural cell membrane coating derived from activated neutrophils is anticipated to camouflage the particles, thereby reducing immune cell ‘attack’ and clearance by macrophages, while conferring a unique ‘affinity’ for the inflammatory foci. Fluorescent images of RAW 264.7 macrophages showed that the amount of endocytosed NM-NP_rhFGF21_ labeled using Texas Red was significantly reduced compared to NP_rhFGF21_. This indicates enhanced immune evasion from macrophage clearance, resulting in a longer circulation half-life for the neutrophil-membrane-coated nanoparticles (Figure 2A). Further, no noticeable cytotoxicity was detected for NM-NP_rhFGF21_ in differentiated cardiomyocyte-like H9C2 cells (Figure S3). Taken together, these results suggest that NM-NP_rhFGF21_ is feasible and may have the advantage of slowing rhFGF21 release while increasing its serum half-life.

### 3.2. Neutrophil Membrane Coating of FGF21 Nanoparticles Facilitates Focal Accumulation at Inflamed I/R Foci

To evaluate the targeting efficiency of NM-NP_rhFGF21_ in vivo, we established an ischemia–reperfusion (I/R) model in mice using a standard surgical approach. NM-NP_rhFGF21_ particles were labeled with Indocyanine Green, a lipophilic fluorescent substance used for identifying anatomical structures and intracorporeal organs, such as the heart, liver, lung, and kidney [25,26]. Near-infrared imaging analysis showed that the ICG-labeled NM-NP_rhFGF21_ had an extended presence in circulation compared to free ICG injected intravenously at the same dose 6 h post I/R operation when imaged at a higher intensity threshold (Figure 2B). Notably, the ICG-labeled NM-NP_rhFGF21_ showed significant accumulation in the I/R-insulted hearts in addition to the liver, compared to uncoated NP_rhFGF21_ when the fluorescence intensity threshold was lowered (Figure 2C), which was only present in the liver as free ICG. The ex vivo fluorescent imaging of the isolated organs showed marked enrichment of ICG-labeled NM-NP_rhFGF21_ in the inflamed I/R heart and liver, which metabolically processed the particles, but not in other organs lacking damage (Figure 2D,E). These data indicate that the neutrophil membrane camouflage prolongs the blood circulation time of NM-NP_rhFGF21_ and confers it the capacity to specifically target inflamed or damaged foci in the heart, potentially enhancing drug efficacy.

### 3.3. NM-NP_rhFGF21_ Retains FGF21 Bioactivity

**Figure 3:**
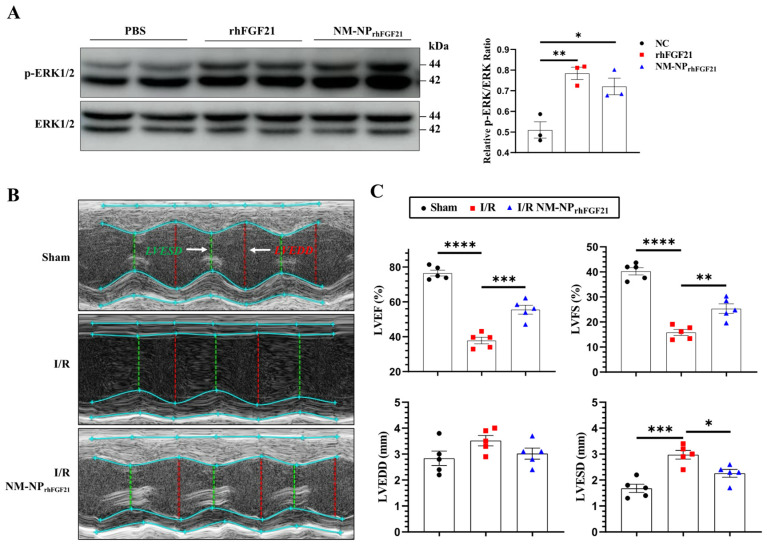
NM-NP_rhFGF21_ improves cardiac function in mice suffering from I/R injury. (**A**) Representative images of Western blot analysis for phospho-ERK1/2 upon stimulation with rhFGF21 and NM-NP_rhFGF21_ in 293T cells transfected with KLB-expressing plasmid. (**B**) Representative M-mode echocardiographic images at day 3 post I/R. White arrow point out annotated line. (**C**) Quantification of LVEF, LVFS, LVESD, and LVEDD. n = 5. Each bar represents the mean ± standard error of the mean (SEM), * *p* < 0.05, ** *p* < 0.01, *** *p* < 0.001, and **** *p* < 0.0001 between two groups.

293T cells, known to express FGFR1, were used to assess the pharmacological activity of NM-NP_rhFGF21_. We compared the activation of the MAPK pathway in 293T cells transfected with the KLB-expressing plasmid. The levels of phospho-ERK1/2, a marker of MAPK pathway activation, stimulated by NM-NP_rhFGF21_ were similar to those stimulated by free rhFGF21 at 100 ng/mL (Figure 3A). These results suggest that rhFGF21 loaded into the NM-NP particles maintains the same level of activity as free rhFGF21 protein.

### 3.4. NM-NP_rhFGF21_ Administration Leads to Enhanced Improvement of Cardiac Function Following I/R Injury

**Figure 4:**
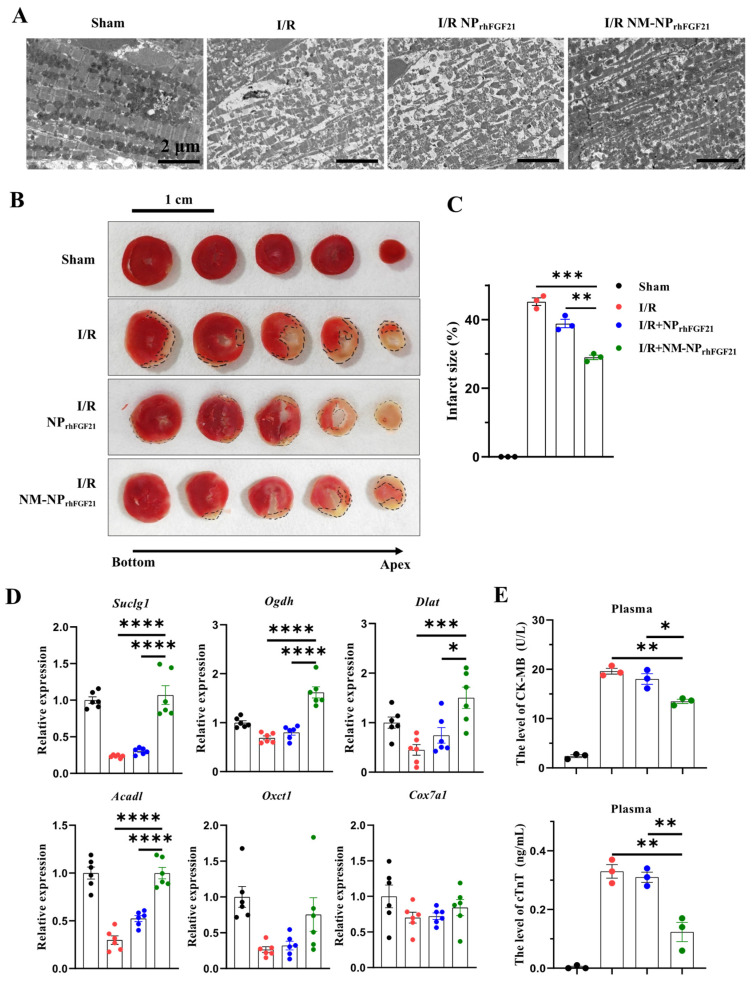
NM-NP_rhFGF21_ reduces cardiac damage in mice suffering from I/R injury by promoting mitochondrial energetic function. (**A**) Representative images of heart sections showing cardiomyocyte mitochondria and myofibrils captured by TEM. Scale bar: 2 μm. (**B**) Representative images of TTC staining of the hearts at day 3 post I/R. Scale bar: 1 cm. Black dotted lines indicate the infarcted areas. (**C**) Quantification of infarct size. n = 3. (**D**) qRT-PCR analysis of expression of cardiac genes involved in mitochondrial energy metabolism including *Suclg1*, *Ogdh*, *Dlat*, *Acadl*, *Oxct1*, and *Cox7a1*. n = 6. (**E**) CK-MB and cTNT levels in plasma. n = 3. Each bar represents the mean ± standard error of the mean (SEM), * *p* < 0.05, ** *p* < 0.01, *** *p* < 0.001 and **** *p* < 0.0001 between two groups.

To assess the pharmacological efficacy of rhFGF21 delivered by NM-NP_rhFGF21_, we analyzed the heart functional parameters using echocardiography 3 days post-I/R. The injection of NM-NP_rhFGF21_ significantly increased the left ventricular ejection fraction and fractional shortening, which were highly reduced following I/R compared to the normal sham control (Figure 3B,C). Transmission electron microscopy revealed that NM-NP_rhFGF21_ treatment significantly restored dense, tightly organized mitochondria intercalated with myocardial fibrils to levels similar to the sham group. This contrasted markedly with the loose, ruptured myocardial fibrils and swollen, dispersed mitochondria in the I/R group treated with the vehicle control (Figure 4A). An analysis of the traverse section of the heart via TTC staining revealed a significant reduction in infarct size following NM-NP_rhFGF21_ treatment compared to the PBS treatment control, as well as the sham and vehicle controls (Figure 4B,C). The expression levels of critical genes involved in mitochondrial energy metabolism, including *Suclg1* (succinate-CoA ligase, GDP-forming, alpha submit), *Ogdh* (oxoglutarate dehydrogenase), *Dlat* (dihydrolipoamide S-acetyltransferase), *Acadl* (acyl-Coenzyme A dehydrogenase, long-chain), *Oxct1* (3-oxoacid CoA transferase 1), and *Cox7a1* (cytochrome c oxidase subunit 7A1), were significantly upregulated in the NM-NP_rhFGF21_-treated group, which was better than that of NP_rhFGF21_ group but not the control group (Figure 4D). Similarly, serum levels of CK-MB (creatine kinase-MB) and cTnT (cardiac troponin T), two cardiac injury biomarkers, were significantly reduced by NM-NP_rhFGF21_ treatment compared to controls (Figure 4E). Taken together, these findings suggest that rhFGF21 delivered by NM-NPs significantly improves the recovery of I/R-damaged hearts compared to noncoated NP_rhFGF21_. This is consistent with other reports showing that neutrophil-membrane-coated nanoparticles more effectively target and alleviate inflammation in diseased or injured tissues [23,27].

### 3.5. NM-NP_rhFGF21_ Administration Leads to Enhanced Reductions in Inflammation and Cardiomyocyte Apoptosis

**Figure 5:**
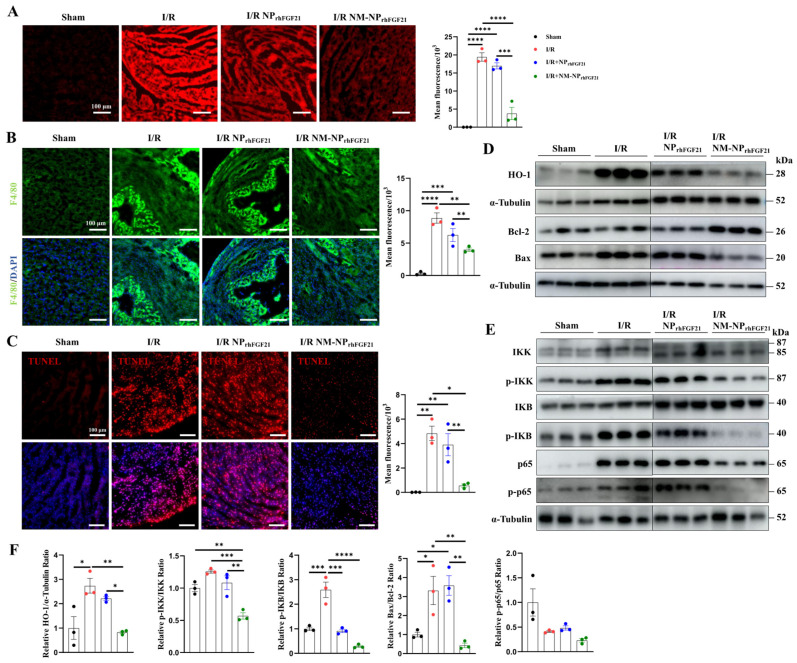
NM-NP_rhFGF21_ improves cardiac function in mice suffering from I/R injury by suppressing oxidative stress, inflammation, and apoptosis. (**A**) Representative fluorescence images of lipidic ROS levels estimated with DCFH-DA fluorescence probe. Scale bar: 100 μm. (**B**) Representative fluorescence images of macrophages stained by anti-F4/80 antibody on heart sections. Green represents macrophages and blue represents nuclei. Scale bar: 100 μm. (**C**) Representative TUNEL staining of heart sections in each group. Red represents TUNEL and blue represents nuclei. Scale bar: 100 μm. (**D**–**F**) Western blot analysis of proteins associated with ROS stress, inflammation, and apoptosis, including HO-1, p65, p-p65, IKK, p-IKK, IKB, p-IKB, Bcl-2, and Bax. Each bar represents the mean ± standard error of the mean (SEM), * *p* < 0.05, ** *p* < 0.01, *** *p* < 0.001, and **** *p* < 0.0001 between two groups.

I/R cardiac injury is characterized by a burst of ROS, which exacerbates ischemic damage. We found that the I/R-injured cardiomyocytes elicited intense ROS, as revealed by ROS-reactive fluorescent staining, compared to normal heart sections. In contrast, the intensity of ROS fluorescence was significantly reduced by NM-NP_rhFGF21_ administration compared to the non-treatment I/R group (Figure 5A). These changes coincided with the levels of HO-1, a marker of ROS generation, as shown by immunoblot analysis (Figure 5D,F).

Recruitment of monocytes and macrophages to the infarcted region contributes to a heightened inflammatory response and excess damage following cardiomyocyte injury [28]. Immunofluorescence staining of F4/80, a macrophage marker, revealed a significantly higher number of macrophages on day 3 post-I/R in hearts, which was markedly reduced by NM-NP_rhFGF21_ administration in the infarcted region (Figure 5B). The macrophage-inhibiting effect of NM-NP_rhFGF21_ was significantly greater than that of NP_rhFGF21_ alone. Correspondingly, the NF-kB inflammatory pathway, marked by levels of p-IKK, p-IKB, p65, and p-p65, was more efficiently attenuated by NM-NP_rhFGF21_ (Figure 5E,F). These results suggest that the targeted delivery of rhFGF21 via NM-NP particles more efficiently subdues inflammation in the I/R foci.

Intense ROS generation and inflammation often cause aggravated cardiomyocyte apoptosis and necrosis [29,30]. TUNEL staining of the heart sections showed reduced cardiomyocyte apoptosis following NM-NP_rhFGF21_ treatment compared to the PBS vehicle control and NP_rhFGF21_ treatment (Figure 5C). The ratio of Bax to Bcl-2, which, respectively, promotes and inhibits the apoptotic pathway [27], was significantly decreased by NM-NP_rhFGF21_ (Figure 5D,F). Altogether, our data suggest that the enhanced efficacy of rhFGF21 enclosed in neutrophil-membrane-coated nanoparticles for treating I/R-damaged hearts may be attributable to the increased focal inhibition of ROS damage, inflammation, and cardiomyocyte death.

## 4. Conclusions and Discussion

**Figure 6:**
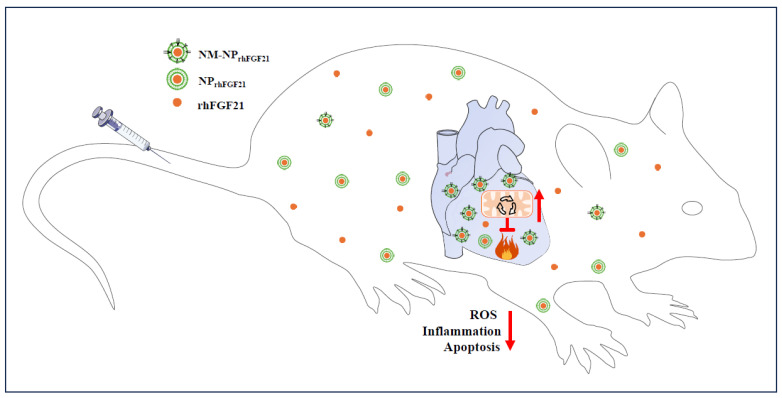
Schematic of the benefits of neutrophil-membrane-coated FGF21 nanoparticles. The neutrophil membrane coating of FGF21 nanoparticles serves not only to camouflage FGF21 nanoparticles, protecting them from macrophage efferocytotic clearance, but also to enhance their accumulation in I/R foci due to neutrophilic attraction to the inflammatory site. The net result is a cascade of positive effects, including reductions in oxidative stress, inflammation, and cardiomyocytic death and the significant enhancement of mitochondrial energetic function, leading to improvements in cardiac performance. It should be noted that the accumulation of NM-NP_rhFGF21_ in the liver (Figure 2B–D), a phenomenon common to injected exogeneous drugs, may also mimic the metabolic effects of endocrine FGF21, such as promoting adipose tissue lipolysis and hepatic ketogenesis and, thereby, metabolic substrate influx to the heart. The red upward arrow indicates an increase and the red downward arrow indicates a decrease.

Acute myocardial infarction is a global health issue with high morbidity and mortality, largely due to the lack of efficacious treatment options and poor targeted drug delivery to the infarcted areas. In this study, we report an approach for the effective delivery of FGF21 using neutrophil-membrane-coated biodegradable nanoparticles to mouse cardiac sites of I/R, which models human AMI. The injected NM-NP_rhFGF21_ exhibits a longer serum half-life and higher targeted enrichment in the I/R injury foci, resulting in the more effective inhibition of ROS damage, inflammation, and apoptotic cardiomyocyte death compared to conventional nanoparticle delivery approaches (Figure 6). Therefore, our study provides proof-of-concept for the focal delivery of FGF21 as an effective treatment for AMI and potentially other heart diseases.

The development of AMI is multifactorial and often associated with cardiometabolic disorders such as atherosclerosis, hyperlipidemia, fatty heart, diabetes, and severe obesity. In recent years, the prevalence of these cardiometabolic diseases has increased substantially in modernized societies, ranking them among the high-risk factors for AMI. Reducing metabolic disorders in patients with heart problems has shown promising clinical outcomes with the introduction of the blockbuster drug semaglutide, a GLP-1 receptor agonist. In a large-cohort, multi-center study of 17,604 patients with pre-existing cardiovascular disease and overweight or obesity but without diabetes, the weekly subcutaneous administration of semaglutide was superior to placebo in reducing the incidence of death from cardiovascular causes, nonfatal myocardial infarction, or nonfatal stroke [31]. In several previous clinical trials involving patients with type 2 diabetes, GLP-1 receptor agonists reduced major adverse cardiovascular events (such as cardiovascular death, myocardial infarction, or stroke), all-cause mortality, hospital admission for heart failure, and kidney problems [32]. FGF21 is another endocrine metabolic regulator that has shown effectiveness against obesity, diabetes, and fatty liver disease in mice [33,34,35]. Some long-acting FGF21 analogs have entered phase 3 trials for patients with NASH and/or fibrosis [35,36], showing significant improvements in markers of liver injury, fibrosis, and glucose and lipid metabolism. In experimental mice, FGF21 and its analogs have demonstrated strong inhibitory activity against cardiomyopathy, heart hypertrophy, atherosclerotic cardiovascular lesions, and ischemic damage [19,37,38,39,40] by normalizing metabolic derangements and associated tissue-damaging effects. This is consistent with our results, which show that the delivery of FGF21 to the I/R foci beyond the liver resulted in reduced ROS stress, inflammation, apoptotic cardiomyocyte death, and tissue necrosis, indicating FGF21 as a candidate therapy for adverse cardiovascular events.

In physiology, FGF21 is a stress-induced hepatokine that acts on many tissues through circulation where its receptor complex FGFR-KLB is present [41,42,43,44]. Its effect can be a combination of systemic and local, primarily due to its lack of matrix retention via heparan sulfate binding in the extracellular space and basement membrane, unlike other heparan sulfate-binding FGFs. Consequently, injected FGF21 and its analogs are highly diffusive and prone to proteolysis, which, under certain circumstances, may reduce its local concentration and effectiveness, necessitating protection and target enrichment strategies. Many materials and approaches have been devised for the in vivo delivery of drugs, including protein biologics [45]. For instance, a perfluoropropane and poly(lactic-co-glycolic acid)-based nanocarrier for FGF21 has been shown to improve the prophylactic treatment of diabetic cardiomyopathy [46]. The benefits of FGF21 on heart diseases have also been recapitulated through the delivery of FGF21-encoding minigenes or mRNA [47,48]. Our study demonstrates that the neutrophil-membrane-coated nanoparticle delivery of FGF21 is more effective than that of uncoated nanoparticles. This method not only prolongs the circulation half-life of rhFGF21 by protecting it from proteolytic cleavage and liver/kidney clearance but also enhances the focal enrichment of FGF21 due to the high affinity and specificity of the neutrophil membrane for the inflammatory foci. Additionally, the neutrophil membrane provides a camouflaging effect, shielding FGF21 from macrophage efferocytotic clearance. Interestingly, a somewhat reversed design using apoptotic cell-membrane-coated nanoparticles for FGF21 delivery to acute lung injury sites has been shown to enhance the macrophage efferocytosis of FGF21. This modulates lung macrophage polarization and suppresses the excessive secretion of pro-inflammatory cytokines, which are critical for acute lung injury [49]. These studies suggest that the enhanced focal delivery and protection of FGF21, as demonstrated in our study, is a promising avenue for further exploration.

Taken together, the findings of our study demonstrate the advantage of using immune-cell-membrane-coated nanoparticles for delivering FGF21 to inflamed and damaged areas in the heart beyond the liver as the major drug-metabolizing site, which entails better efficacy in reducing inflammation, oxidative stress, cell apoptosis, and necrotic damage compared to noncoated nanoparticles, leading to improved cardiac function after I/R injury (Figure 6). This approach could potentially be used to enhance the targeted delivery of not only FGF21, but also other therapeutic drugs to pathological lesions.

## Figures and Tables

**Table 1:** qRT-PCR primers.

Gene Name	Species	Primer Sequence
*Hprt1*	Mouse	F: 5′-CAGTCCCAGCGTCGTGATTA-3′ R: 5′-TGGCCTCCCATCTCCTTCAT-3′
*Suclg1*	Mouse	F: 5′-GTCTTACACAGCCTCTCGGAAAC-3′ R: 5′-ACTCCAAAGCCTGCTGACTGTG-3′
*Ogdh*	Mouse	F: 5′-GGTGTCGTCAATCAGCCTGAGT-3′ R: 5′-ATCCAGCCAGTGCTTGATGTGC-3′
*Dlat*	Mouse	F: 5′-GCTGCAAACAGCAGAGCTAA-3′ R: 5′-CGCCTCGTTCACCATTTCTC-3′
*Oxct1*	Mouse	F: 5′-CTGGAGTTTGAGGACGGCAT-3′ R: 5′-TCCGCATCAGCTTCGTCTTT-3′
*Acadl*	Mouse	F: 5′-CATTGGTGGGGACTTGCTCT-3′ R: 5′-TGGCTATGGCACCGATACAC-3′
*Cox7a1*	Mouse	F: 5′-ATCCGGAGTCTTAGAACAGGT-3′ R: 5′-CATTCCCCCGCCTTTCAAGT-3′

## Data Availability

The raw data supporting the conclusions of this article will be made available by the authors on request.

## Supplementary Materials

The following supporting information can be downloaded at: https://www.mdpi.com/article/10.3390/nano15050346/s1, Figure S1: Schematic for the experimental concept and procedure; Figure S2: Time-dependent change in the size of NM-NPrhFGF21; Figure S3: Cytotoxicity of NM-NPrhFGF21.
