# Supplementary material for: Enhanced FGF21 Delivery via Neutrophil-Membrane-Coated Nanoparticles Improves Therapeutic Efficacy for Myocardial Ischemia–Reperfusion Injury
Source: Nanomaterials (Basel). 2025 Feb 23;15(5):346. doi: 10.3390/nano15050346 (PMC11901824; doi:10.3390/nano15050346)
Supplement: Supplementary file 1 [file nanomaterials-15-00346-s001.zip › nanomaterials-3440996-supplementary.pdf]

SUPPLEMENTAL INFORMATION

TITLE

Enhanced FGF21 Delivery via Neutrophil-Membrane-Coated Nanoparticles Improves Therapeutic Efficacy for Myocardial Ischemia–Reperfusion Injury

CONTENT

1. SUPPLEMENTAL FIGURES AND LEGENDS.

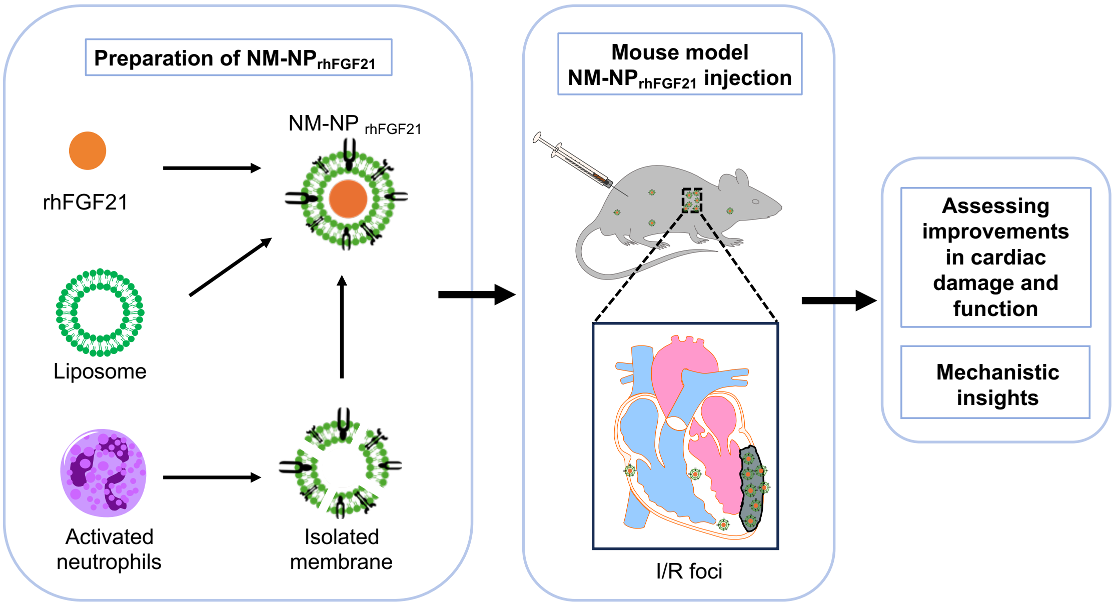

Figure S1. Schematic for the experimental concept and procedure.

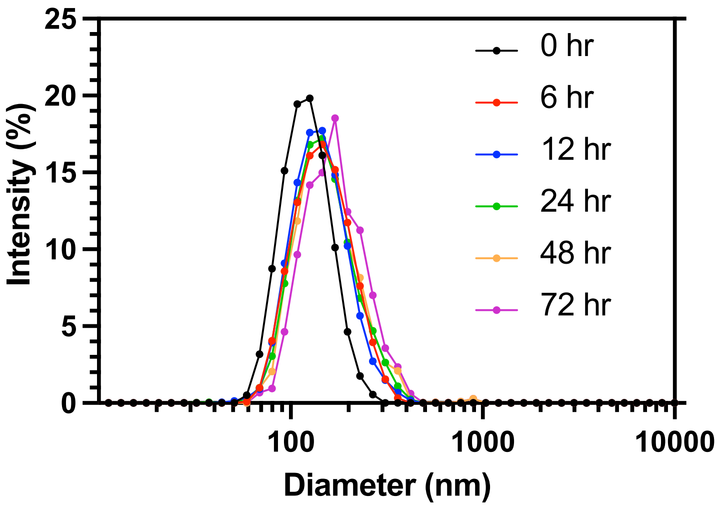

Figure S2. Time-dependent change in the size of NM-NP$_{rhFGF21}$. Aliquots of NM-NPrhFGF21 nanoparticles in cell culture medium containing 10% FBS, which simulates circulation conditions, were monitored at 0, 6, 12, 24, 48, and 72 hours by dynamic light scattering. The hydrodynamic diameters (nm) of the particles were calculated using the Stokes-Einstein equation, which were plotted against intensities to visualize the size distribution of the particles in the sample over time.

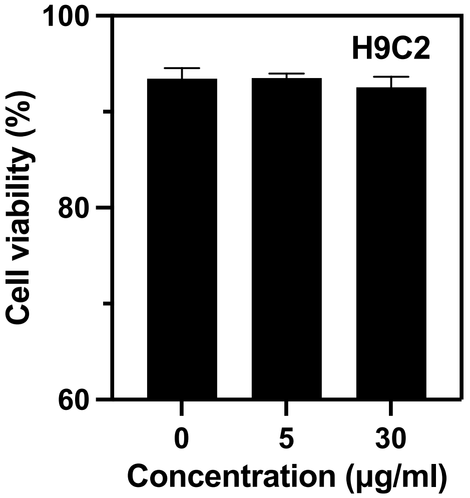

Figure S3. Cytotoxicity of NM-NP$_{rhFGF21}$. Aliquots of NM-NP$_{rhFGF21}$ nanoparticles at 0, 5, and 30 μg/ml protein concentrations were used to treat the differentiated H9C2 cells in culture. A CCK8 assay was then performed to analyze cell viability as described in Materials and Methods.
